# Machine learning-based identification of lactate metabolism-associated biomarkers in non-alcoholic fatty liver disease

**DOI:** 10.1007/s10238-026-02250-z

**Published:** 2026-07-21

**Authors:** Xiaocui Wang, Xinhan Wang, Liang Zhao, Li Song

**Affiliations:** 1 College of Traditional Chinese Medicine, Ningxia Medical University, Yinchuan Ningxia, 750004 China; 2 Key Laboratory of Dryness Syndrome in Chinese Medicine, Ministry of Education, Ningxia Medical University, Yinchuan Ningxia, 750004 China; 3 Ningxia Regional Key Laboratory of Integrated Traditional Chinese and Western Medicine for Prevention and Treatment of Regional High Incidence Disease, Ningxia Medical University, Yinchuan Ningxia, 750004 China; 4 College of Basic Medicine, Ningxia Medical University, Yinchuan Ningxia, 750004 China; 5 Department of Nephrology, Affiliated Hospital of Traditional Chinese Medicine, Ningxia Medical University, Yinchuan Ningxia, 750001 China; 6 Present Address: College of Traditional Chinese Medicine, Ningxia Medical University, No. 1160, Shengli Street, Xingqing District, Yinchuan City, Ningxia Hui Autonomous Region China

**Keywords:** Non-alcoholic fatty liver disease, Lactate metabolism, Machine learning, Weighted gene co-expression network, Biomarker

## Abstract

To determine lactate metabolism-associated biomarkers for non-alcoholic fatty liver disease (NAFLD). Based on NAFLD datasets from the gene expression omnibus database and lactate metabolism-related genes from GeneCards database, NAFLD-lactate metabolism hub genes (NAFLD-LM HGs) were screened via differential analysis, weighted gene co-expression network analysis and four machine learning algorithms. Their diagnostic efficacy was evaluated; molecular subtyping was performed; single-cell RNA sequencing (scRNA-seq) was subsequently conducted; and validation was finally conducted in NAFLD mouse models. We finally screened out eight NAFLD-LM HGs, namely CCAAT/enhancer-binding protein alpha (CEBPA), flavin containing dimethylaniline monooxygenase 1 (FMO1), insulin-like growth factor-binding protein 1 (IGFBP1), krüppel-like factor 4 (KLF4), low-density lipoprotein receptor (LDLR), myelocytomatosis oncogene (MYC), nuclear receptor subfamily 4 group A member 2 (NR4A2), and thymidylate synthase (TYMS), with a diagnostic rate of 0.798 and an area under the curve of 0.948. These genes drove the molecular heterogeneity in NAFLD patients by modulating of metabolism and immune microenvironment. The results of scRNA-seq clarified that Th17 cells were identified as the most abundant annotated cell subset. Through animal experiments, five genes (LDLR, MYC, IGFBP1, NR4A2, and KLF4) were established to have potential protective effects against NAFLD. Five genes (LDLR, MYC, IGFBP1, NR4A2, KLF4) are identified as “protective factors”, and their downregulation is associated with NAFLD progression. The remaining three genes (CEBPA, FMO1, TYMS) exhibit inconsistent expression patterns, suggesting bidirectional regulation.

**Supplementary Information:**

The online version contains supplementary material available at https://doi.org/10.1007/s10238-026-02250-z.

## Background

Non-alcoholic fatty liver disease (NAFLD) is a frequent driver of chronic hepatopathy globally [1]. In terms of hepatocyte injury, NAFLD includes a series of disease spanning from milder forms such as non-alcoholic fatty liver (NAFL) to more serious forms including non-alcoholic steatohepatitis (NASH), hepatic fibrosis, cirrhosis, and even liver failure and hepatocellular carcinoma (HCC) [2]. NAFLD frequently coexists with one or more risk factors of cardiac metabolism. Hence, it has garnered considerable attention and is also called metabolic dysfunction-associated steatotic liver disease (MASLD) [3].

Lactate is a byproduct of glycolysis. The liver, as the primary site of gluconeogenesis, maintains net lactate uptake and the highest net lactate clearance rate [4]. In NAFLD, metabolic disturbances in hepatocytes include enhanced glycolysis and elevated lactate levels [5]. Lactate is involved in the onset of NAFLD primarily through monocarboxylate transporters (MCTs; MCT1: transporting lactate into the cell; MCT4: transporting lactate out of the cell) and G protein-coupled receptor 81 (GPR81, a lactate receptor) [4]. MCT1 haploinsufficient mice on a high-fat diet are effectively resistant to the development of hepatic steatosis [6]. GPR81 is downregulated in the livers of obese mice, which increases hepatic lipid accumulation and exacerbates hepatic steatosis [7]. Additionally, lactate can facilitate the onset of NAFLD, liver fibrosis, and even HCC by modulating various biological processes through lactylation (a post-translational modification), such as inflammatory responses, immune cell function, energy metabolism, and the activation of hepatic stellate cells (HSCs) [8, 9]. In summary, dysregulation of lactate generation and transport, as well as lactylation, is closely linked to NAFLD, representing a potentially promising direction for future research.

**Fig. 1:**
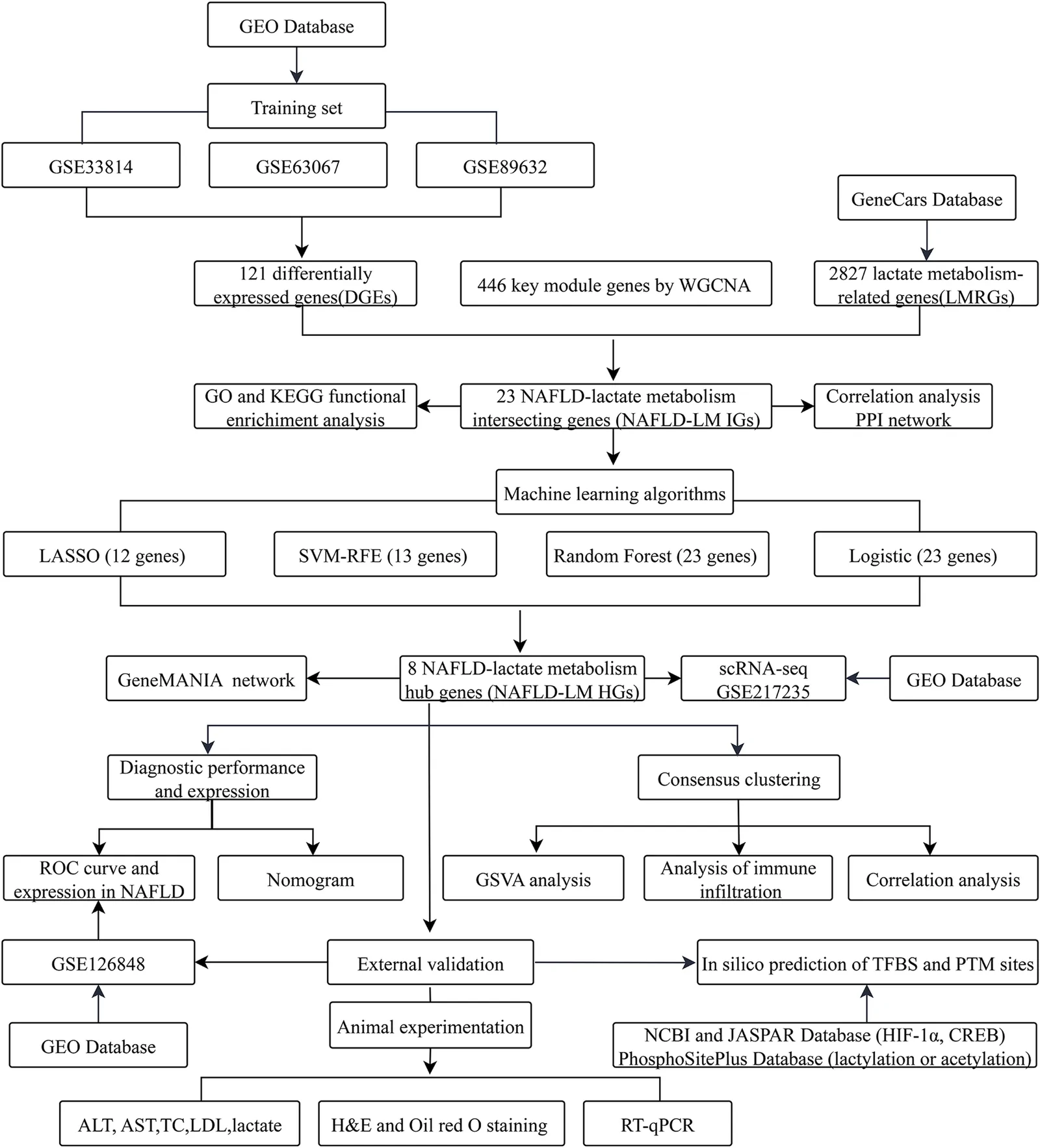
Flowchart of data analysis

Thus, this study intended to identify biomarkers closely related to lactate metabolism in NAFLD by merging weighted gene co-expression network analysis (WGCNA) with four machine learning algorithms, thereby presenting novel ideas for the assessment and intervention of NAFLD. The workflow for data analysis of this study is detailed in Fig. 1 (more detailed and specific analytical procedures are provided in Figure S1).

## Methods

### Data acquisition and processing

The gene expression omnibus (GEO) (https://www.ncbi.nlm.nih.gov/geo/) is a publicly accessible repository for functional genomics data. In GEO, “NAFLD” and “Homo sapiens” were adopted as search terms and the “GEOquery” package (information related to R packages is provided in Table S1) was employed to download five datasets, namely GSE33814 [10], GSE63067 [11], GSE89632 [12], GSE126848 [13], and GSE217235 [14]. The first three datasets were integrated using the “sva” package (ComBat function) to remove batch effects. The merged dataset acted as a training set, while GSE126848 served as a validation set. GSE217235 was employed for single-cell sequencing analysis (basic information for all datasets provided in Table S2).

We used “lactate metabolism” as the search term and applied a screening threshold of relevance score > 5. A total of 2,827 lactate metabolism-related genes (LMRGs; Table S3) were ultimately acquired from the GeneCards database (v5.26; https://www.genecards.org/).

### Screening of differential expression genes (DEGs)

Differential analysis for NAFLD and control samples in the training set was carried out leveraging “limma”. DEGs were extracted using the screening conditions of adjusted *P* < 0.05 and |log_2_ fold change (FC)| > 0.585. The Benjamini-Hochberg method was applied to control the false discovery rate. The “ggplot2” and “pheatmap” packages were used to generate a volcano plot and a heatmap, respectively.

### WGCNA

WGCNA, as a systematic biology method, was applied to describe gene associations among distinct samples. It was employed to identify gene modules with similar expressions and to analyze their relations with sample features [15].

The “WGCNA” package was employed to perform WGCNA on the training set with the first 3,000 genes ranked by median absolute deviation. The main steps were as follows. (1) Hierarchical clustering was performed to remove outlier samples. (2) We selected an appropriate soft-thresholding power (β) based on R² = 0.9 and the highest value at which mean connectivity approached zero to construct scale-free networks. (3) Modules were partitioned via the dynamic tree cut algorithm (minModuleSize = 50, mergeCutHeight = 0.25). (4) Similar modules were merged based on the dissimilarity of module eigengenes (CutHeight = 0.1). (5) Pearson correlation analysis was used to evaluate correlations between modules and traits, thereby identifying key module genes strongly linked to traits (*P* < 0.05).

### Acquisition, enrichment analysis, correlation analysis, and protein-protein interaction (PPI) network construction of NAFLD-lactate metabolism intersecting genes (NAFLD-LM IGs)

Key module genes acquired from WGCNA were intersected with DEGs and LMRGs. A Venn diagram of the intersecting genes was constructed with the “ggvenn” package, thereby obtaining NAFLD-LM IGs.

Gene Ontology (GO) and Kyoto Encyclopedia of Genes and Genomes (KEGG) enrichment analyses were performed leveraging the database for annotation, visualization and integrated discovery (DAVID) (https://davidbioinformatics.nih.gov/home.jsp). *P* < 0.05 was considered statistically significant. The first 10 enriched terms, sorted by gene count, were selected for visualization. A pathway‑gene network diagram was plotted using the SRplot online platform [16] (https://bioinformatics.com.cn/srplot).

Spearman correlation analysis was performed on NAFLD-LM IGs using the “linkET” package, and the results were presented. *P* < 0.05 was considered statistically significant.

The PPI network was developed leveraging the search tool for the retrieval of interacting genes/proteins (STRING) database (medium confidence: 0.4) (v12.0; https://cn.string-db.org/). Afterwards, this network was presented with Cytoscape software (v3.10.3), where node sizes were set according to their “degree” values.

### Machine learning and the development of the co-expression gene network

Based on NAFLD-LM IGs, four machine learning algorithms, namely least absolute shrinkage and selection operator (LASSO) regression, support vector machine-recursive feature elimination (SVM-RFE), random forest (RF), and logistic regression (LR), were applied to further select NAFLD-lactate metabolism hub genes (NAFLD-LM HGs).

LASSO screened variables by adding a penalty term during model estimation. In this process, the regression coefficients of some non‑essential variables were compressed to zero, thereby eliminating them from the model [17, 18]. LASSO regression was executed using “glmnet”. The optimum value of lambda was established via 5‑fold cross‑validation for gene screening. SVM‑RFE, as an efficient feature selection technique, iteratively removed features with the lowest weights, thereby identifying key hub genes [19]. SVM‑RFE was carried out with the “e1071” and “caret” packages. RF, as an ensemble learning method composed of decision trees, trained and predicted samples by constructing hierarchical tree‑like structures [20]. The “randomForest” package was used with “ntree” set to 1,000. The RF model was built using the number of trees with the smallest error, and the significance of genes in the model was evaluated. Genes with a value of importance > 0.1 were selected. LR, also known as the logistic model, can link results (dependent variables) to predictor variables (independent variables) [21]. This supervised learning method facilitated the selection of relevant features by weighting them [22]. A univariate LR model was constructed with the “glm” function from the “stats” package, and forest plots were generated with the “forestplot” package. Finally, a Venn diagram was created for the genes screened by these four machine learning algorithms to obtain NAFLD-LM HGs.

A co‑expression gene network based on NAFLD-LM HGs was constructed using the gene multiple association network integration algorithm (GeneMANIA) website (https://genemania.org/) to predict gene functions.

### Construction and validation of the receiver operating characteristic (ROC) curves and nomogram

The ROC curves were employed to evaluate the utility of classification models. The area under the curve (AUC) was adopted to identify test precision. A higher AUC corresponded to better accuracy [23]. To assess the diagnostic utility of NAFLD-LM HGs for NAFLD in the training set, we plotted ROC curves and calculated AUCs using “pROC” and “ggplot2” packages. This assessment was further validated with an external dataset. The expression levels of NAFLD‑LM HGs were compared between the NAFLD group and the control group using the Wilcoxon rank‑sum test and presented with box plots.

A nomogram model for NAFLD-LM HGs was constructed with the “rms” and “regplot” packages to forecast the occurrence probability of NAFLD. The model was subsequently validated and evaluated with a calibration plot. The “rmda” package was utilized to plot decision curves to appraise the clinical net benefit of patients.

### Consensus clustering and gene set variation analysis (GSVA)

To evaluate the potential biological functions of NAFLD-LM HGs in NAFLD, we performed consensus clustering analysis on 81 NAFLD samples in the training set. Consensus clustering was carried out with the “ConsensusClusterPlus” package. This statistical and visualization method, based on a resampling approach, integrated multiple clustering results to construct a consensus matrix. It was leveraged to confirm the count of sample clusters and to appraise the coherence and credibility of the findings of clustering [24]. The k‑means algorithm was adopted with Kmax = 10 and “Euclidean Distance” was selected as the distance metric [25]. By examining the cumulative distribution function (CDF) and the area under the CDF curves for different k values, we selected the most stable k [24], thereby dividing the samples into distinct clusters.

DEGs between different clusters were identified using the “limma” package, with the screening criteria of adjusted *P* < 0.05 and |log_2_ FC| > 0.585. A volcano plot was subsequently generated.

GSVA was conducted to uncover the biological mechanisms underlying different clusters of individuals with NAFLD [26]. First, gene sets for GO and KEGG were downloaded from the molecular signatures database (MSigDB; https://www.gsea-msigdb.org/gsea/msigdb/) using the “msigdbr” package. Subsequently, by applying “GSVA” with the “Gaussian” algorithm, we generated a sample-by-gene-set score matrix. Differential pathways were then extracted and presented.

### Immune infiltration and analysis for correlation between immune cells and NAFLD-LM HGs

The cell-type identification by estimating relative subsets of ribose nucleic acid (RNA) transcripts (CIBERSORT) algorithm accurately identified and estimated the relative ratios of multiple immune cell subsets from gene profiles of complicated tissues. It incorporated the LM22 dataset [27], including a gene matrix defining 22 immune cell types. By leveraging the “CIBERSORT” package, we estimated the levels of infiltration of these 22 kinds of immune cells across distinct clusters. Subsequently, Spearman analysis was implemented to appraise the relations of NAFLD-LM HGs with the 22 immune cell types.

### Single-cell RNA sequencing (scRNA-seq) and expressions of NAFLD-LM HGs across cell subsets

The scRNA-seq dataset GSE217235 included three NAFLD samples (GSM6709882, GSM6709883, and GSM6709885), and it was analyzed using the “Seurat” package. The analysis procedure consisted of the following steps. The 10× scRNA-seq data of each sample were read to construct a Seurat object. Quality control and filtering were performed by setting thresholds based on the number of genes expressed (nFeature_RNA) and the proportion of mitochondrial genes (percent.mt). After data normalization, 2,000 highly variable genes (HVGs; Table S4) were established using the “vst” method. Subsequently, dimensionality reduction was carried out via principal component analysis (PCA), batch effects among samples were removed leveraging the “Harmony” algorithm, and unsupervised clustering was conducted. The results were visualized using uniform manifold approximation and projection (UMAP). Finally, manual cell annotation was carried out based on the signature genes of hepatic infiltrating T helper cell (CD4^+^) subsets of NAFLD [14, 28], and the specific expression patterns of NAFLD-LM HGs in each cell subset were displayed.

### In silico prediction of NAFLD-LM HGs transcription factor binding sites (TFBS) and corresponding protein post-translational modification (PTM) sites

We used bioinformatics approaches to elucidate the potential indirect association between NAFLD‑LM HGs and lactate metabolism. First, we obtained the promoter analysis regions of these genes from the National Center for Biotechnology Information (NCBI) database (https://www.ncbi.nlm.nih.gov/; “Homo sapiens” was selected, and sequences spanning 2000 base pairs upstream of the transcription start site of each gene were extracted). The JASPAR database (https://jaspar.elixir.no/; species was set to “Homo sapiens”; relative profile score threshold was set to 80% by default) was then used to predict whether hypoxia-inducible factor 1-alpha (HIF-1α) and cAMP-responsive element-binding protein (CREB) binding sites were present in the promoter regions of these genes. Additionally, the PhosphoSitePlus database (v6.8.2; https://www.phosphosite.org) was used to retrieve previously reported lactylation or acetylation modification sites on the proteins encoded by these genes.

### Establishment of NAFLD mouse models and histopathological examination

Ten C57BL/6J mice, aged between 6 ~ 8 weeks, were acclimatized for one week and then randomized into a control group and an NAFLD group (*n* = 5 each). Mice in the model group were fed a high‑fat diet (45% fat Kcal%). Control mice received a standard chow diet (11.1% fat Kcal%). All mice were housed under room temperature and standard lighting conditions and fed continuously for 22 weeks, with body weight measured weekly. After 22 weeks, all animals were fasted for 12 h, liver weight was measured, and the liver index (liver weight/body weight) was calculated. Blood was collected from orbits and centrifuged to obtain serum. Serum lipid parameters (total cholesterol (TC), low density lipoprotein (LDL)) and liver function markers (alanine aminotransferase (ALT) and aspartate aminotransferase (AST)) were measured using a fully automated biochemical analyzer (Hitachi 7180, Japan). Serum lactate concentration was measured with a lactate assay kit (Jiancheng, Nanjing, China). Hematoxylin and eosin (H&E) and oil red O staining was carried out to reveal pathological alterations in the liver tissues of NAFLD mice. Fresh liver tissues were immersed in 4% formaldehyde, encased in paraffin, and divided into 4 μm slices for H&E staining. For oil red O staining, frozen liver tissues were rewarmed, encased in the optimal cutting temperature compound, and cut into 10 μm cryosections. These cryosections were subsequently stained with oil red O solution in a closed system and counterstained with hematoxylin.

### Real-time quantitative polymerase chain reaction (RT-qPCR)

RNA was extracted from liver tissues using Trizol. Complementary deoxyribonucleic acid (cDNA) was prepared using a reverse transcription kit (SparkJade, Shandong, China). Primers for the NAFLD-LM HGs were provided by Ningxia Saisi Juxin Biotechnology Co., Ltd. (Table S5). RT-qPCR was performed leveraging an ABI 7500 real-time PCR detection system and SYBR Green qPCR Mix (SparkJade, Shandong, China). Both the reverse transcription and the preparation of RT-qPCR reaction mixtures were carried out on ice. β-actin was used as an internal reference gene, and relative gene expression was validated using the 2^−ΔΔCt^ method.

### Statistical analysis

Bioinformatics analyses and figure generation were performed using R (v4.4.2; https://www.r-project.org/). ROC curve analysis was executed to appraise the diagnosis utility of hub genes for NAFLD. Pearson’s method was adopted to examine correlations of modules with traits. Spearman’s method was applied to analyze the relation between genes and that of immune cells with genes. For the animal experiments, GraphPad Prism (v10.4.1) was adopted for analysis and graphing. Measured data were depicted as the mean ± standard deviation (SD). If data were normally distributed and satisfied homogeneity of variance, intergroup differences were evaluated employing the unpaired two-tailed Student’s t-test. For data with heterogeneity of variance, Welch’s corrected t-test was applied. The Mann-Whitney U test was adopted for data non-normally distributed. *P* < 0.05 was considered statistically significant.

## Results

### Determination of DEGs

**Fig. 2:**
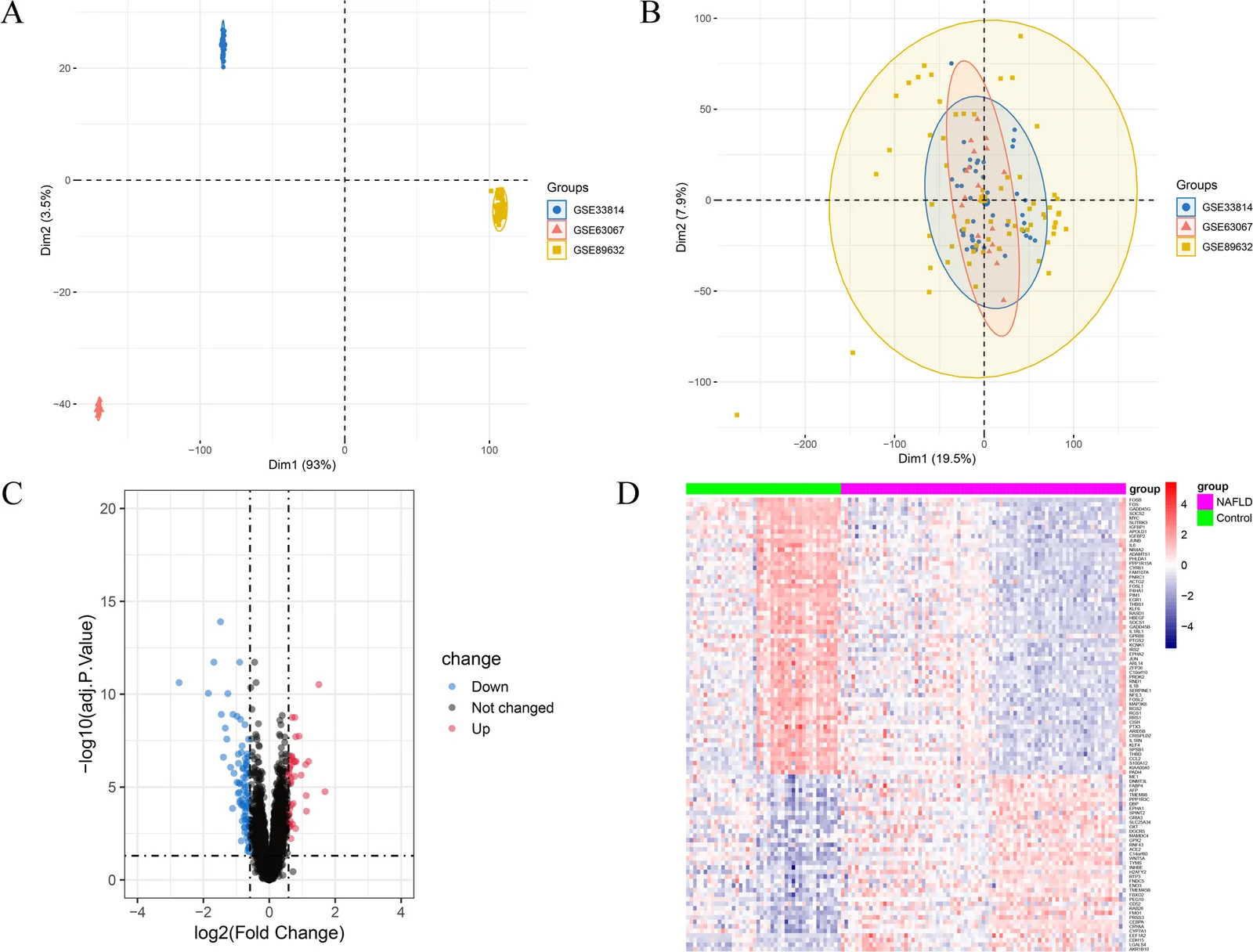
Data processing results of the training set and screening of DEGs between the NAFLD group and the control group. PCA plot for samples before (**A**) and after (**B**) the correction of batch effects. (**C**) Volcano plot of DEGs (39 upregulated and 82 downregulated). (**D**) Heatmap of DEGs (Top 50 upregulated and top 50 downregulated genes. Each row represents a gene, and each column denotes a sample; “red” (high expression) and “blue” (low expression) represent gene expression levels)

A total of 125 samples (81 NAFLD and 44 controls) were acquired from three training datasets, including GSE33814, GSE63067 and GSE89632. After the correction of batch effects, samples from distinct datasets clustered together more closely in the PCA space, and systematic differences between batches were significantly reduced. These indicated an effective control of batch effects (Fig. 2A and B). Differential analysis identified 121 DEGs (Table S6) in NAFLD, with 39 upregulated and 82 downregulated (Fig. 2C and D).

### A total of 446 key module genes identified by WGCNA

**Fig. 3:**
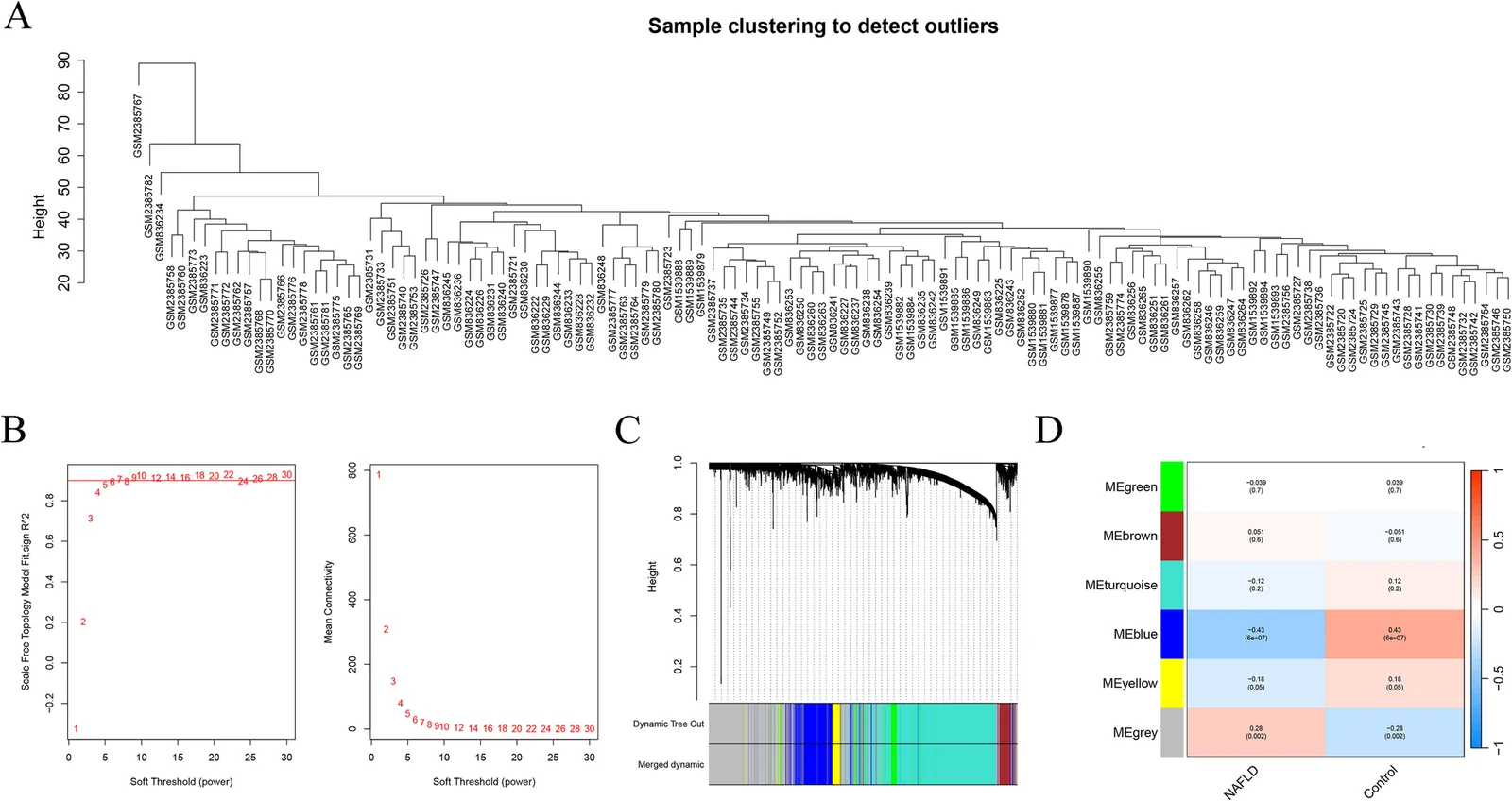
Development of WGCNA and recognition of key genes. (**A**) Hierarchical clustering dendrogram of samples. (**B**) Establishment of the optimal soft‑thresholding power for constructing a scale‑free network (For the “Scale Free Topology Model Fit”, i.e., “signed R²”, a higher value indicates that the network more closely approximates a scale‑free network. “Mean connectivity” represents the average connectivity of the network under different soft thresholds). (**C**) Clustering dendrogram of WGCNA co‑expression network modules based on dissimilarity (Resulting in 6 gene modules). (**D**) Heatmap for correlations between modules and traits (“Red” (positive correlation) and “blue” (negative correlation) stand as correlations, with darker colors indicating stronger correlations)

No outliers were detected in the training set (Fig. 3A). Scale-free networks were constructed by selecting β = 10 (R^2^ = 0.9) (Fig. 3B). After similar modules were merged, eight gene modules were identified (Fig. 3C). Among those modules, only the blue one was significantly related to NAFLD (correlation coefficient (r) = -0.43, *P* = 6e-07) (Fig. 3D). Consequently, 446 key module genes (Table S7) were extracted from this module.

### Enrichment analysis, correlation analysis, and PPI network of 23 NAFLD-LM IGs

**Fig. 4:**
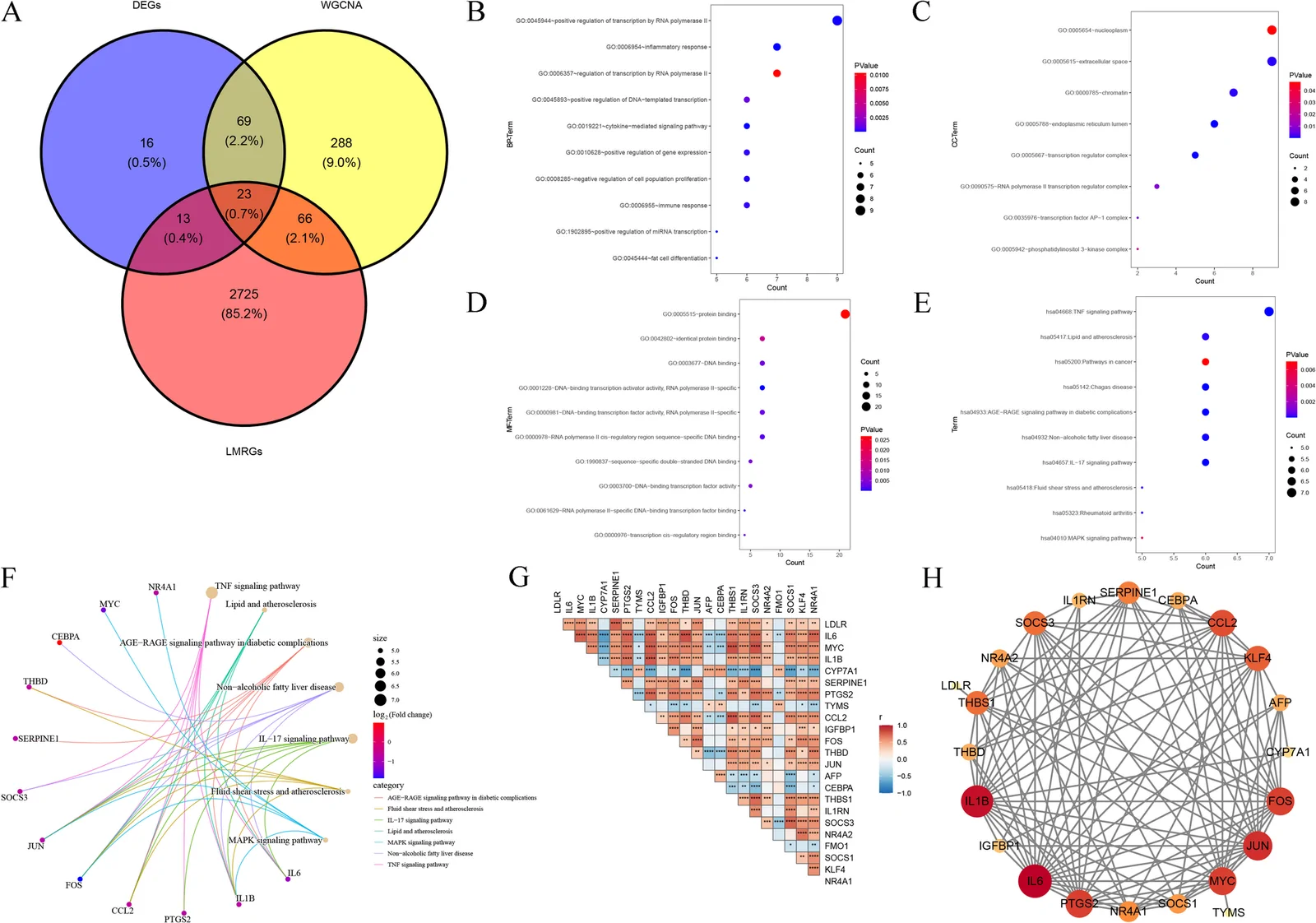
Collection and analysis of NAFLD-LM IGs. (**A**) Venn diagram for the 23 intersecting genes among DEGs, genes from WGCNA, and LMRGs. (**B**-**E**) GO and KEGG functional enrichment analysis of 23 NAFLD-LM IGs (The first 10 terms ranked by gene count are shown). (**F**) Network diagram for the relationships between some KEGG pathways and their enriched genes (The color of each gene represents its expression trend (“Red”: upregulation, “blue”: downregulation). The node size of each pathway denotes the number of genes enriched in that pathway). (**G**) Correlation heatmap for the 23 NAFLD-LM IGs (“Red” denotes positive correlation and “blue” negative correlation, with darker colors indicating stronger correlations). (**H**) PPI network of the 22 NAFLD-LM IGs (FMO1, an isolated gene, has been automatically excluded from the network. Node size and color intensity represent the degree). *P < 0.05, ** *P* < 0.01, *** *P* < 0.001, **** *P* < 0.0001

After 446 module genes were intersected with 121 DEGs and 2,827 LMRGs, 23 NAFLD-LM IGs (Table S8) were acquired (Fig. 4A). GO enrichment analysis yielded 78 biological process (BP) terms, 8 cellular component (CC) terms, and 20 molecular function (MF) terms (Tables S9-S11). The first 10 terms ranked by count value were selected for visualization (Fig. 4B-D). Among the 39 KEGG enrichment pathways (Table S12), the tumor necrosis factor signaling pathway, lipid and atherosclerosis pathway, and non-alcoholic fatty liver disease pathway were significantly enriched by these genes (Fig. 4E and F). In summary, these 23 intersecting genes encompassed various biological functions such as metabolism, inflammation, immunity, cell proliferation, and differentiation, and they were correlated with each other at varying degrees (Fig. 4G (Table S13), 4 H).

### NAFLD-LM HGs identified by four machine learning methods and their function prediction

In total, 12 and 13 genes were identified from the 23 NAFLD-LM IGs using the LASSO regression algorithm (Fig. 5A and B; Table S14) and the SVM-RFE algorithm (Fig. 5C; Table S15), respectively. Neither the RF algorithm nor the LR model eliminated any genes, thus retaining all 23 genes (Fig. 5D and E (Table S16), 5 F (Table S17)). After the genes acquired from the four machine learning methods were intersected, eight NAFLD-LM HGs were ultimately determined, namely CCAAT/enhancer-binding protein alpha (CEBPA), flavin containing dimethylaniline monooxygenase 1 (FMO1), insulin-like growth factor-binding protein 1 (IGFBP1), krüppel-like factor 4 (KLF4), low-density lipoprotein receptor (LDLR), myelocytomatosis oncogene (MYC), nuclear receptor subfamily 4 group A member 2 (NR4A2), and thymidylate synthase (TYMS) (Fig. 5G; basic information is provided in Table S18). Among them, MYC was related to the stress-activated protein kinase signaling cascade pathway, LDLR was implicated in the negative regulation of external stimuli and the regulation of lipid metabolism, and KLF4 was linked to the negative regulation of cell migration and external stimuli (Fig. 5H).

**Fi. 5g:**
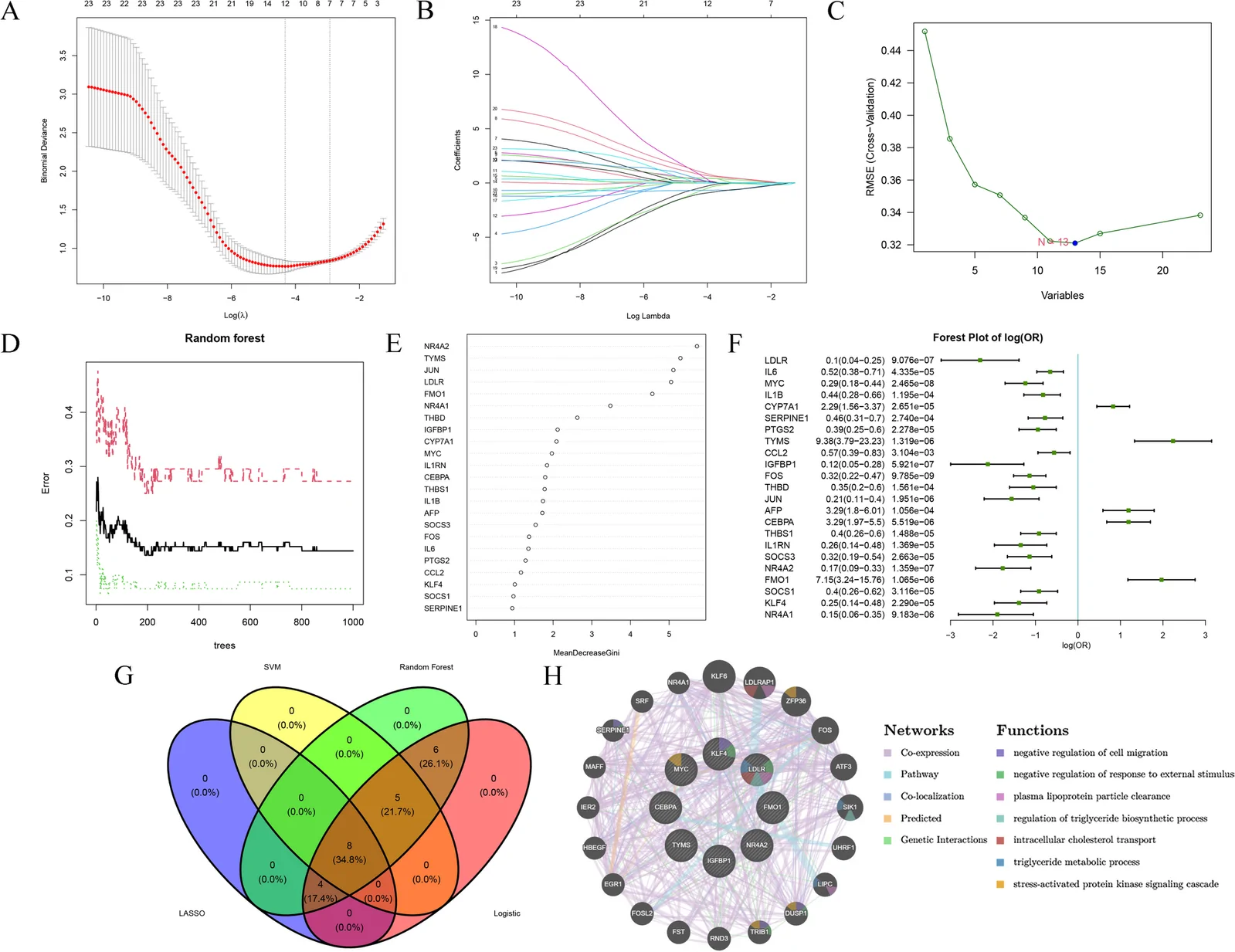
Further screening of NAFLD-LM HGs utilizing machine learning and establishment of a gene co‑expression network. (**A**, **B**) In total, 12 genes identified by the LASSO regression algorithm (A: Cross‑validation curve. The y‑axis represents the cross‑validation error (lower values indicate better model fit), and the numbers above the x‑axis represent the number of genes retained in the model under the corresponding λ. B: Coefficient path plot. A path plot showing how gene coefficients are gradually shrunk to zero as λ changes). (**C**) A total of 13 genes identified by the SVM‑RFE algorithm (The x‑axis denotes the number of genes (23 in total), and the y‑axis represents the root mean square error (RMSE) of cross‑validation. The model error is minimized in the case of *N* = 13). (**D**, **E**) The 23 genes analyzed by the random forest algorithm (D: Model error rate for trees 1-500, lower values indicate a more stable model; E: Bubble plot of importance scores for the 23 genes). (**F**) The 23 genes evaluated by the logistic regression model (The x‑axis represents “log (OR)”. Green squares indicate the regression effect values, black horizontal lines represent the 95% confidence intervals (CIs), and the blue vertical line indicates the line of no effect. The corresponding OR (95% CI) and P‑value for each gene are listed on the left). (**G**) Venn diagram for the 8 NAFLD‑LM HGs identified by the four machine learning algorithms. (**H**) Co‑expression network based on the 8 NAFLD‑LM HGs

### Diagnostic and predictive performance of eight NAFLD-LM HGs

**Fig. 6:**
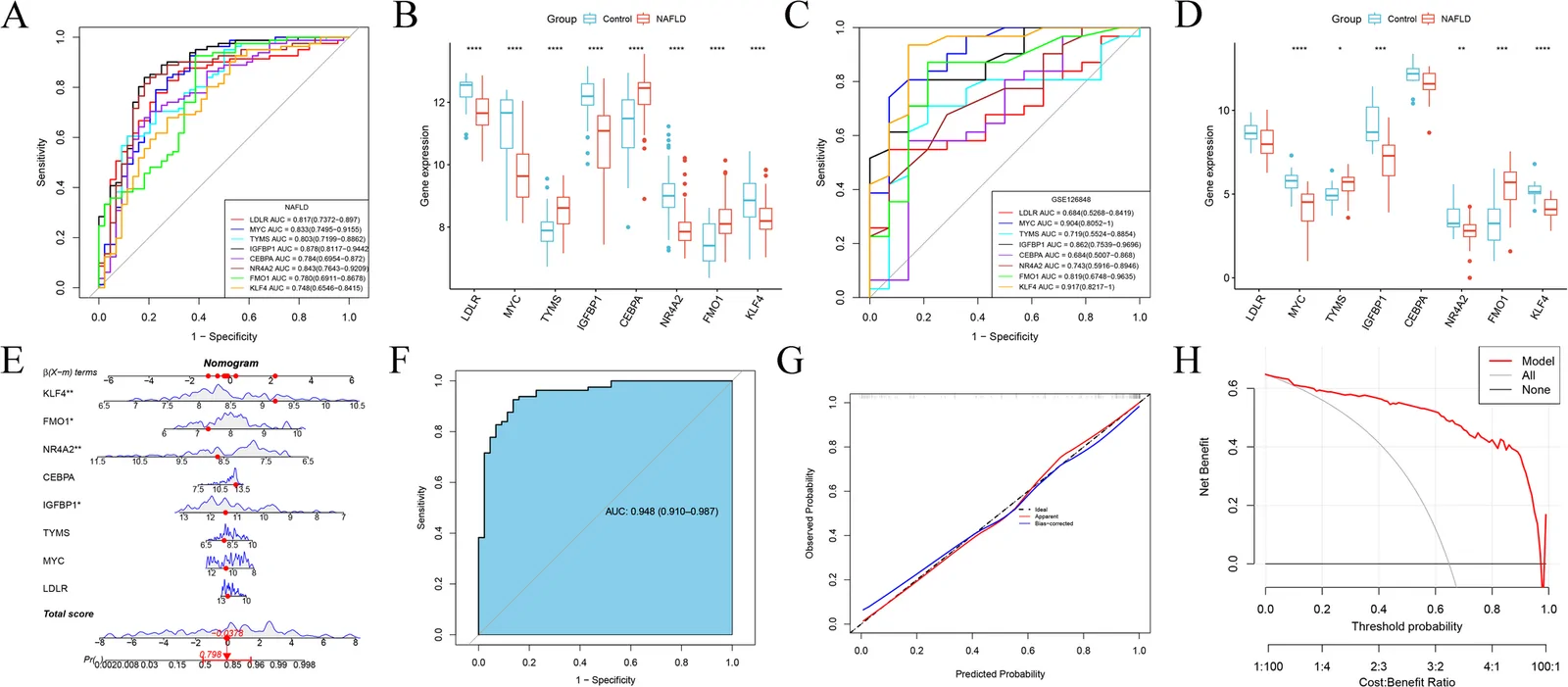
Construction and validation of ROC curves and nomograms based on the eight NAFLD-LM HGs. (**A**) ROC curves for the eight NAFLD-LM HGs in the training set (The x‑axis represents the false positive rate; lower values indicate more accurate exclusion of negative samples by the model. The y‑axis represents the true positive rate; higher values indicate that the classifier can more accurately predict positive samples. The larger the AUC, the stronger the predictive ability of the gene). (**B**) Expression box plots for comparisons of NAFLD-LM HGs between the NAFLD group and the control group in the training set (The central line represents the median, the box represents the interquartile range (IQR), the whiskers extend to 1.5 times the IQR, and points beyond the whiskers are considered outliers). (**C**) ROC curves for the eight NAFLD-LM HGs in the validation set. (**D**) Expression box plots for comparisons of NAFLD-LM HGs between the NAFLD group and the control group in the validation set. (**E**) Nomograms for the eight NAFLD-LM HGs (Each row represents a gene. The blue curve shows the distribution of the gene’s values across samples, and the red dot indicates the value for the current sample. Based on the value of each gene, a corresponding score is obtained; these scores are summed to give the “Total score”, which is used to predict the probability of disease occurrence [Pr()]). (**F**) ROC curve for validation of the eight NAFLD-LM HGs. (**G**) Calibration curves for the nomogram-based diagnostic model (The x‑axis and y‑axis represent the theoretical risk predicted by the model and the actual probability of occurrence, respectively. The closer the solid blue line is to the black dashed line, the stronger the calibration ability of the model). (**H**) Decision curves for the nomogram-based diagnostic model (The threshold probability represents the psychological acceptance threshold for intervention by clinicians or patients. The solid red line is the prediction curve of the model based on the eight hub genes, representing the net benefit when deciding to intervene according to the risk probability predicted by the model. The solid gray line represents the net benefit assuming that all patients receive the intervention. When the solid red line is above the solid gray line, it indicates that patients obtain a net benefit). *P < 0.05, ** *P* < 0.01, *** *P* < 0.001, **** *P* < 0.0001

In the training set, all eight NAFLD-LM HGs had AUCs greater than 0.7. Among them, LDLR, MYC, TYMS, IGFBP1, and NR4A2 possessed better diagnostic performance for NAFLD (AUCs > 0.8) (Fig. 6A). The intergroup comparison indicated that LDLR, MYC, IGFBP1, NR4A2, and KLF4 were downregulated in the NAFLD group (Fig. 6B (Table S19)), suggesting that they may be protective genes against NAFLD. These results were further verified in the GSE126848 dataset (Fig. 6C and D (Table S20). The eight NAFLD-LM HGs collectively achieved a diagnostic rate of 0.798 for NAFLD (Fig. 6E) and had stronger predictive power (AUC = 0.948) (Fig. 6F). The calibration curve reflected that the nomogram was well calibrated, confirming the reliability of the results (Fig. 6G). Decision curve analysis showed that using the nomogram for NAFLD risk assessment in clinical practice yielded a good net benefit (Fig. 6H).

### Consensus clustering, GSVA, immune infiltration, and analysis for relations of immune cells with genes

**Fig. 7:**
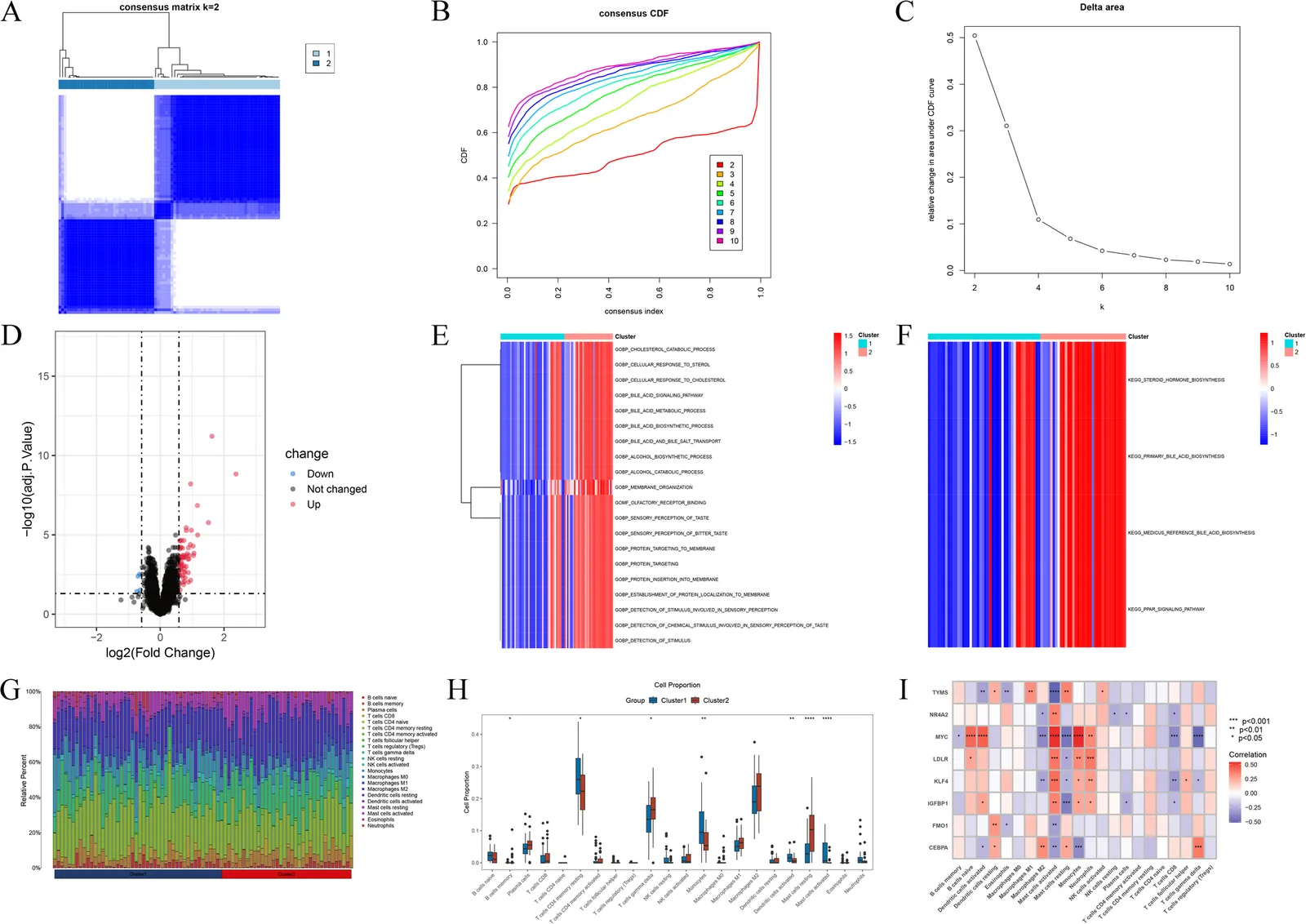
Consensus clustering, GSVA, immune infiltration, and analysis for the relation of immune cells with genes of 81 NAFLD samples based on the eight NAFLD-LM HGs. (**A**) Consensus clustering matrix at k = 2 (Cluster 1 containing 46 samples, Cluster 2 containing 35 samples). (**B**) Consensus CDF curves at k = 2-10 (Curves with different colors show the CDF distribution for different numbers of clusters (k). The closer the curve approaches an ideal step‑shape, the more stable and reliable the clustering effect is for that corresponding k value). (**C**) Relative change in area under the CDF curves (The x‑axis represents the number of clusters (k), and the y‑axis the relative change in the area under the CDF curve between adjacent k values). (**D**) 69 DEGs between the two clusters (65 upregulated and 4 downregulated). (**E**, **F**) GO functional (Only the first 20 GO-BP terms are shown) and KEGG pathway enrichment analysis of the 69 DEGs between the two clusters by GSVA. (**G**) Distribution proportion of the 22 kinds of immune cells in every individual sample across the two clusters. (**H**) Analysis of infiltration for the 22 classes of immune cells across the two clusters. (**I**) Correlations between the 8 NAFLD-LM HGs and the 22 categories of immune cells (“Red” denotes positive correlation and “blue” negative correlation, with darker colors indicating stronger correlations). *P < 0.05, **P < 0.01, ***P < 0.001, ****P < 0.0001

Based on the expression profiles of NAFLD-LM HGs, consensus clustering analysis was performed on 81 NAFLD samples. The clustering was most stable at k = 2 (Fig. 7A-C), yielding 69 DEGs (Fig. 7D; Table S21). The GSVA scores for all functions and pathways in the two clusters were calculated. 78 GO terms (Table S22) and 4 KEGG terms (Table S23) were obtained respectively through differential analysis, and these terms were then presented. The GO-BP terms were related to the following functional modules: cholesterol and bile acids (BAs) metabolism, alcohols metabolism, protein targeting and membrane insertion, and sensory perception and signal detection (Fig. 7E). The KEGG terms involved the biosynthesis of BAs and steroid hormones, as well as peroxisome proliferator activated receptors (PPARs)-mediated lipid metabolism (Fig. 7F). In summary, the expression patterns of NAFLD-LM HGs differed between the two clusters of individuals with NAFLD. The highly activated metabolic programs and perceptive functions were the core biological characteristics of Cluster 2.

The distribution proportion of immune cells per sample across the two clusters was also examined (Fig. 7G) and the infiltration degrees of immune cells were appraised (Fig. 7H; Table S24). Notably, T cells CD4 memory resting, monocytes, dendritic cells activated, and mast cells (MCs) activated were substantially infiltrated in Cluster 1, while T cells gamma delta and MCs resting were remarkably infiltrated in Cluster 2. This suggested that the two clusters exhibited different types of immune responses. Specifically, cluster 1 tended toward adaptive immune initiation and inflammatory activation, while cluster 2 tended toward innate immunity and maintenance of tissue homeostasis. MCs activated were substantially correlated with all eight NAFLD-LM HGs (positively related to NR4A2, MYC, LDLR, KLF4, and IGFBP1; negatively linked to TYMS, FMO1, and CEBPA). Compared with other genes, MYC showed the most extensive correlations with immune cells (positively correlated with B cells naive, dendritic cells activated, MCs activated, monocytes, and neutrophils, and negatively related to B cells memory, macrophages M2, MCs resting, T cells CD8, and T cells gamma delta) (Fig. 7I; Table S25).

### Single-cell sequencing and analysis

**Fig. 8:**
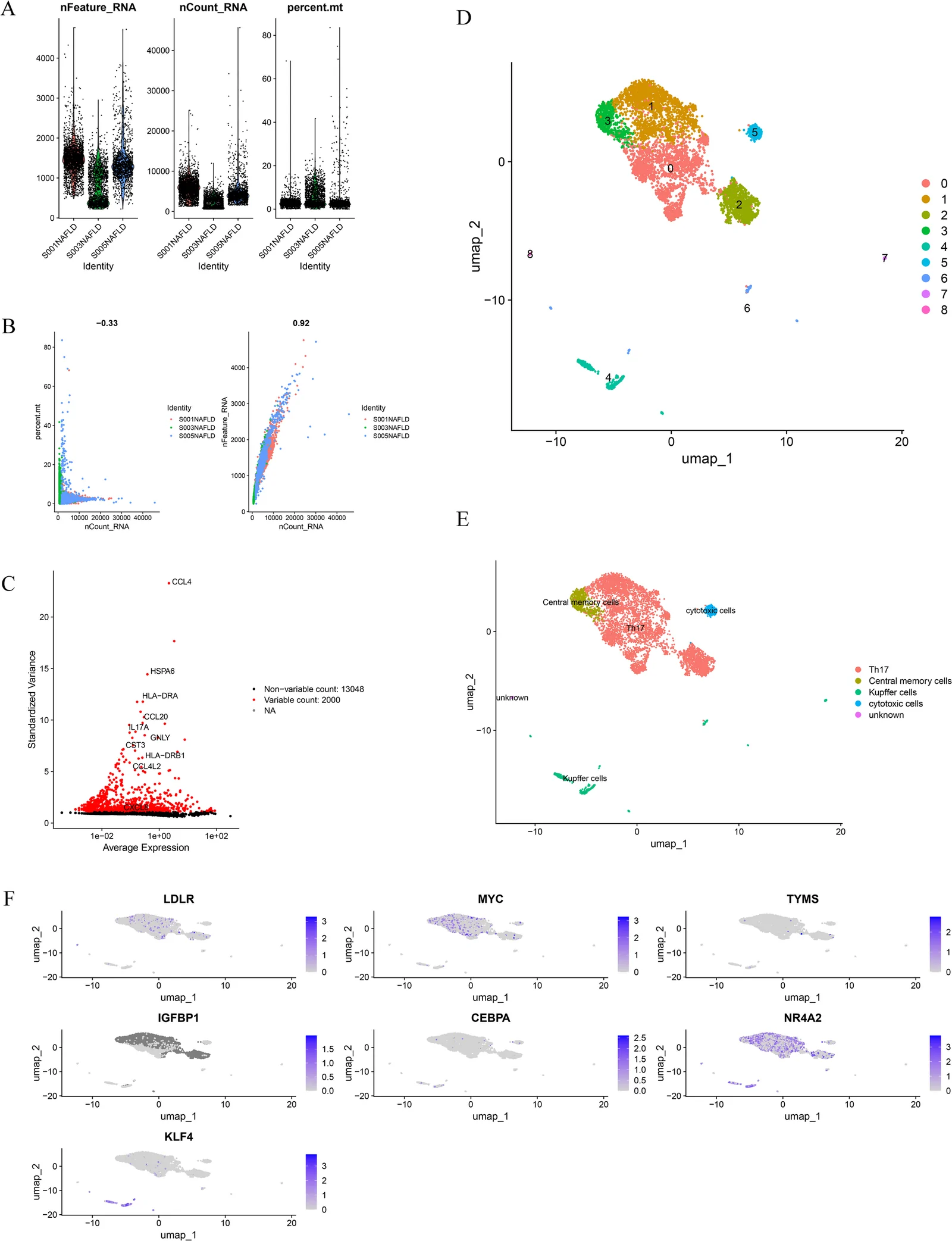
scRNA-seq, annotation, and gene mapping in the GSE217235 dataset. (**A**) Distribution plots for parameters of quality control for scRNA-seq of three NAFLD samples (Including the count of feature genes, sequencing depth, and mitochondrial gene percentage). (**B**) Correlations of sequencing depth with both mitochondrial genome and the total number of intracellular feature genes. (**C**) Visualization scatter plot of the top 10 HVGs. (**D**) Results of UMAP dimensionality reduction and clustering (All cells from the three NAFLD samples clustered into nine distinct clusters. Each dot in the figure represents a single cell). (**E**) Cell types identified with CD4^+^ T cell marker genes. (**F**) Expression of the seven NAFLD-LM HGs across different cell subsets (FMO1 not expressed in any cell subset. “ Blue” represents the gene expression level, with darker colors indicating higher expression of the gene in the corresponding cell subset)

A total of 5,746 cells were identified from three NAFLD samples in the scRNA-seq dataset (GSE217235). Based on the sample quality control plot (Fig. 8A), cells with nFeature_RNA between 200 and 3,000 and percent.mt < 20 were retained. After further filtration of abnormal cells, 5,619 cells were ultimately acquired for subsequent analysis. A negative relationship was observed between sequencing depth and the mitochondrial gene sequence (*r* = -0.33), while a strong positive relationship existed between sequencing depth and the total count of feature genes per cell (*r* = 0.92) (Fig. 8B). Among the 2,000 HVGs, the top 10 expressed genes were presented (Fig. 8C), and nine distinct cell clusters were established (Fig. 8D). Through annotation with CD4^+^ T cell marker genes, four immune cell subsets were ultimately defined. Among them, T helper cell 17 (Th17) was the largest subset (Fig. 8E). LDLR was primarily expressed in Th17, MYC was mainly expressed in central memory cells and Th17, NR4A2 was expressed across all four subsets, and KLF4 was primarily expressed in Kupffer cells (KCs). TYMS and CEBPA were scattered across four cell clusters, while IGFBP1 was not expressed in any cell cluster (Fig. 8F). FMO1, as a metabolic enzyme gene, primarily catalyzed the oxidative metabolism of nitrogen- and sulfur-containing exogenous substances [29], and its expression was driven by a hepatocyte-specific promoter [30] and was highest in hepatocytes [31]. Therefore, it was not expressed in immune cells.

### In silico prediction of TFBS and PTM sites for CEBPA, FMO1, LDLR, and TYMS

We used the JASPAR and PhosphoSitePlus databases to predict molecular evidence supporting an indirect association of CEBPA, FMO1, LDLR, and TYMS with lactate metabolism. JASPAR analysis revealed high‑score binding sites for HIF‑1α and CREB in the promoter regions of all four genes (with binding scores > 5.0) (Tables S26-S29). No lactylation sites were retrieved from PhosphoSitePlus for these genes. However, lysine acetylation sites were identified in CEBPA and FMO1 (Table S30).

### In vivo experimental validation of eight NAFLD-LM HGs

**Fig. 9:**
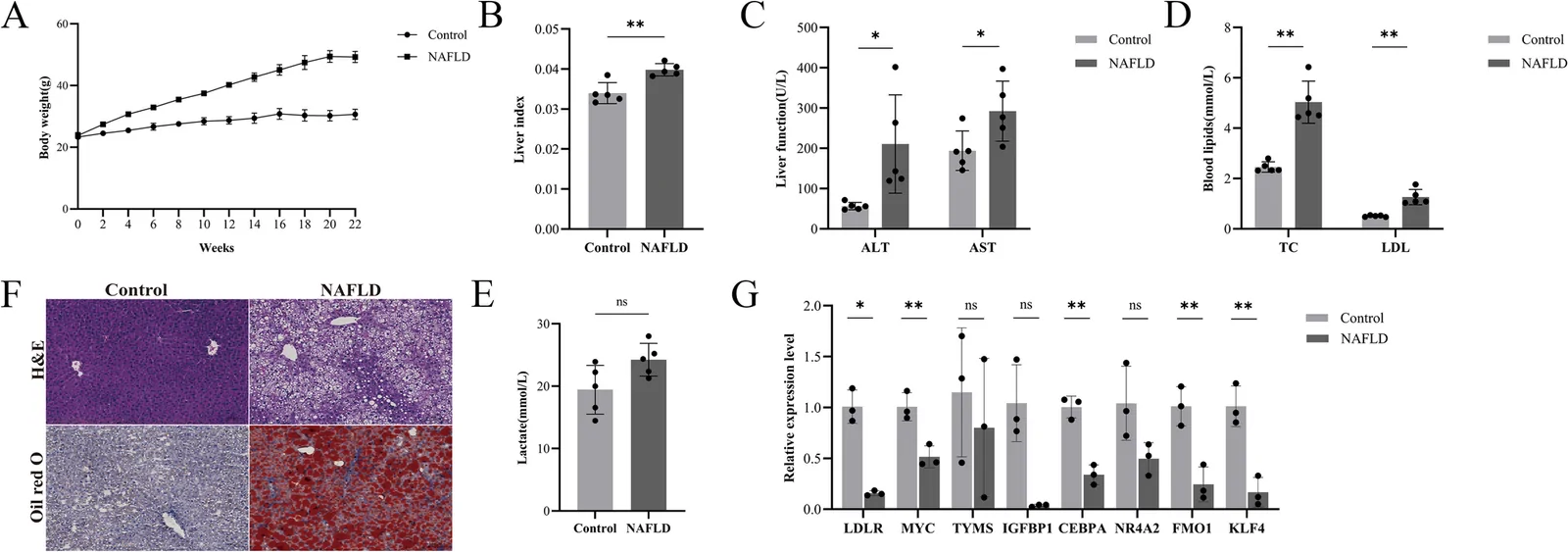
In vivo experimental validation of the eight NAFLD-LM HGs. (**A**) Trend of body weight gain of mice in the control group and the NAFLD group (*n* = 5, each). (**B**) liver index of mice in the control group and the NAFLD group (*n* = 5, each). (**C**, **D**, **E**) Serum concentrations of TC, LDL, ALT, AST and lactate of mice in the control group and the NAFLD group (*n* = 5, each). (**F**) H&E and oil red O staining of livers from mice in the control group and the NAFLD group (scale bar: 100 μm). (**G**) Relative expressions of hub genes in liver tissues of mice in the control group and the NAFLD group validated with RT‑qPCR (*n* = 3, each, three biological replicates). Data are presented as mean ± SD. Statistical analysis was performed utilizing student’s t-test, Welch’s corrected t-test or Mann-Whitney U test (Table S31). *P < 0.05, **P < 0.01; “ns” indicates no statistically significant difference (*P* > 0.05)

After 22 weeks of feeding with a 45% high-fat diet, significant increases were found in body weight and liver index of NAFLD mice (Fig. 9A and B). Levels of serum TC, LDL, ALT, AST, and lactate were elevated (Fig. 9C-E). Hepatic lobular architectures were disordered, inflammatory cells were infiltrated in hepatic sinusoids, and numerous lipid droplets were diffusely distributed in hepatocyte cytoplasm (Fig. 9F). These findings revealed that NAFLD mice had liver tissue damage, lipid deposition, and reduced lactate utilization efficiency. Compared with those in control mice, all hub genes were downregulated in liver tissues of NAFLD mice (Fig. 9G).

## Discussion

The multiple-hit pathogenesis, including insulin resistance (IR), chronic inflammation, oxidative stress, lipid peroxidation, energy metabolism disorders, and epigenetic factors, has been used to explain the complex pathological mechanisms underlying the onset and progression of NAFLD [32]. The liver is a key organ that controls systemic energy metabolism and supplies energy for cellular activities through glycolysis (the conversion of glucose to pyruvate in the liver) [33]. Lactate dehydrogenase (LDH) is an oxidoreductase composed of two subunits, H and M, and mediates the interconversion of pyruvate and lactate via LDH-M (LDHA) and LDH-H [5, 33]. Hepatic metabolic reprogramming, particularly the activation of the glycolytic pathway, is an important driver of the progression from NAFL to inflammation, fibrosis, and even HCC, and is also a cause of lactate accumulation [33].

This study ultimately identified eight NAFLD-LM HGs (CEBPA, FMO1, IGFBP1, KLF4, LDLR, MYC, NR4A2, and TYMS). Among them, the genes directly associated with lactate metabolism are MYC, KLF4, IGFBP1, and NR4A2. Cellular MYC (c-MYC) is a core member of the MYC family [34]. c-MYC overexpression is involved in lactate metabolism by activating key glycolytic genes (e.g., phosphofructokinase, LDHA) [35–37] and upregulating the expression of lactate transmembrane transporters (MCT1/MCT4) [38, 39]. In a breast cancer model, KLF4 overexpression activates glycolysis and increases lactate production [40]. However, other studies have shown that KLF4 overexpression limits lactate production and uptake by reducing glycolytic flux, negatively regulating LDHA expression [41], and inducing MCT4 expression [42]. Compared with wild-type mice, transgenic mice with IGFBP1 overexpression exhibit significantly reduced serum lactate levels, although the regulatory mechanism remains unclear [43]. NR4A2 is a potent transcriptional activator of glycolysis in the mouse heart, and LDHA overexpression leads to increased lactate content [44]. Thus, these genes are primarily involved in lactate metabolism through the regulation of glycolysis or lactate transporters.

Although there is a lack of literature evidence supporting the direct association of CEBPA, FMO1, LDLR, and TYMS with lactate metabolism, we predicted molecular evidence of their indirect association using the JASPAR and PhosphoSitePlus databases. The JASPAR predictions showed that binding motifs for HIF‑1α and CREB were present in the promoter regions of CEBPA, FMO1, LDLR, and TYMS. Under hypoxic conditions, HIF‑1α maintains glycolysis and lactate production by promoting LDHA expression, and simultaneously regulates MCTs activity to mediate lactate efflux from cells [45, 46]. In human epidermal growth factor receptor 2-transfected cells, CREB directly affects lactate production and intracellular acidification by regulating glycolysis, particularly LDH [47]. Based on these findings, we hypothesized that CEBPA, FMO1, LDLR, and TYMS might participate in lactate metabolic reprogramming through HIF‑1α‑ and CREB‑mediated transcriptional regulation. To further search for evidence of lactylation and acetylation sites related to lactate metabolism in these genes, we retrieved the PhosphoSitePlus database. We did not directly retrieve lactylation sites for CEBPA, FMO1, LDLR, or TYMS from PhosphoSitePlus. This may be related to the fact that lactylation research is still in its early stages [48, 49] and that these proteins have not yet been specifically included in lactylation proteomics analyses. Therefore, it does not imply that these genes undergo no lactylation and are completely unrelated to lactate metabolism. We identified acetylation sites in CEBPA and FMO1. Lactylation and acetylation share lysine modification sites, and their regulatory functions in gene transcription and metabolic regulation are somewhat similar, possibly relying on the same regulatory proteins, namely “Writers”, “Erasers”, and “Readers” [49]. Most proteins that are lactylated also undergo acetylation [49]. Therefore, the identified acetylation sites on CEBPA and FMO1 may also be potential lactylation candidate sites, thereby linking these genes to lactate metabolism. This speculation, however, remains to be further validated experimentally. In summary, the predictions from JASPAR and PhosphoSitePlus provide indirect evidence for the association of CEBPA, FMO1, LDLR, and TYMS with lactate metabolism at the levels of TFBS and PTM, respectively, and offer important clues for subsequent in‑depth functional studies of these genes.

We identified five genes (LDLR, MYC, IGFBP1, NR4A2, and KLF4) that were downregulated in both NAFLD patients and mouse liver tissues. This suggests that they may be potential protective genes against NAFLD. This finding is consistent with previous studies: these low-expressed genes can be involved in the progression of NAFLD through various mechanisms, like promoting lipid synthesis, enhancing IR, and facilitating extracellular matrix deposition [50–54].

However, conflicting results have been reported in other studies. Under inflammatory conditions in NAFLD, LDLR expression is upregulated and promotes excessive accumulation of cholesterol [55]. c-MYC overexpression can drive the progression of MASLD through multiple mechanisms, encompassing promoting glycolysis, lipid synthesis, and oxidative stress, and activating HSCs [56]. Overexpression of NR4A2 and KLF4 is associated with pro-inflammatory effects in NAFLD [57, 58]. Notably, one study has suggested that the upregulation of IGFBP1 in NAFLD may represent a compensatory response of the body, resulting from complex regulation of IGFBP1 phosphorylation, to counteract lipid accumulation and inflammation [59]. In our study, the expression trends of the other three genes (CEBPA, FMO1, TYMS) diverge between NAFLD patients and mouse liver tissues (upregulated in patients but showing downregulated mRNA expression levels in mouse livers). In different studies, CEBPA expression can be upregulated by various metabolic factors like IR, inflammation, and endoplasmic reticulum stress [60], or can promote liver fibrosis through downregulation [61]. In a NAFLD cell model, overexpressed FMO1 inhibits PPARα (a subtype of PPARs) expression and upregulates ferroptosis, leading to dysregulated lipid metabolism [62]. However, a contradictory study has demonstrated that in a high-fat diet-induced rat fatty liver model, FMO1 expression and activity are downregulated [63]. The discrepancy may arise from: (1) cell-type specificity: the cellular model employs a specific hepatocyte cell line, whereas the animal model comprises multiple intrahepatic cell populations; (2) compensatory feedback: high-fat diet may downregulate FMO1 via nuclear receptors such as pregnane X receptor/constitutive androstane receptor as an adaptive response [63]; (3) dose-effect relationship: differential outcomes between overexpression and physiological downregulation. Upregulation of TYMS in NAFLD has been confirmed by multiple studies [64–66] and may be related to mitochondrial dysfunction affecting hepatic energy metabolism [67].

Based on existing research evidence (including our study), we observed opposite expression trends of these eight hub genes in NAFLD. This suggests that these genes may have the potential for bidirectional regulation in NAFLD, and the expression direction of the same gene is changed in the case of different disease stages, metabolic microenvironments, or individual genetic backgrounds. Currently, the mechanisms by which these hub genes directly regulate lactate metabolism pathways to influence the NAFLD progression remain unclear. However, given that genes like MYC have been demonstrated to regulate glycolytic metabolic flux and the expression of lactate transporters, this clue provides a feasible starting point for further in-depth investigation.

Consensus clustering results of NAFLD patients indicated that the eight hub genes drove the molecular heterogeneity of NAFLD patients through regulating the synthesis of BAs and steroid hormones, and lipid metabolism. BAs are key end metabolites derived from cholesterol breakdown in hepatocytes and can be classified into primary (synthesized in the liver) and secondary BAs (produced by gut microbiota through the conversion of primary BAs) [68]. Individuals with MASLD exhibit excessive BAs, abnormal signaling, and BAs metabolic dysregulation in the gut [69], which are closely associated with hepatic steatosis, hepatocyte swelling, and liver fibrosis [70]. BAs can directly affect LDH activity, but the effect varies depending on the BAs species, conjugation status, and target cell characteristics [71]. PPARs are key transcription factors that regulate lipid metabolic homeostasis and include three subtypes, namely α, β, and γ [72]. They are closely involved in multiple pathological processes during the NAFLD progression, including dysregulation of lipid and energy metabolism, oxidative stress, and inflammatory stimulation, and PPARs agonists can ameliorate these pathological processes [72]. The impact of PPARs on lactate metabolism differs among cell types. In adipocytes, PPARα agonists inhibit glycolysis and limit lactate production [73]. In contrast, in HepG2 cells, PPARγ agonists promote glycolysis and lactate production [74]. This differential effect may be due to distinct metabolic programs executed under different cellular metabolic states. Steroid hormones are divided into four categories, including glucocorticoids, and have multiple biological functions like the regulation of metabolism [75]. Cholesterol is the main raw material for steroid hormone synthesis [76]. Steroid hormones influence hepatic steatosis, inflammation, and fibrosis by regulating lipid metabolism‑related genes [75]. Cortisol (the main glucocorticoid in humans) helps activate the activity of enzymes related to gluconeogenesis (the process of synthesizing glucose from non‑carbohydrate substances like lactate [4]) and promotes the conversion of lactate into glycogen [73]. In summary, BAs, PPARs, and steroid hormones are involved in lactate metabolism. They not only share cholesterol as a common precursor but also exhibit extensive cross‑regulation among themselves [75, 77], collectively participating in the pathological progression of NAFLD. These metabolic pathways were activated in cluster 2 (Fig. 7F). According to its immune infiltration profile (Fig. 7H), this cluster is characterized by metabolic adaptation (e.g., PPARs activation) and innate immune homeostasis. This suggests that individuals in this cluster may be in an early or slowly progressive stage of NAFLD. In contrast, cluster 1 features metabolic decompensation (e.g., PPARs inhibition) and adaptive immune‑driven inflammatory activation. This result indicates that individuals in this cluster may be more consistent with active NASH.

The liver is a vital immune organ. The interaction between innate and adaptive immune cells promotes the occurrence and progression of NAFLD [78]. Based on the interaction patterns between the eight hub genes and immune cells, the biological roles of these genes were summarized into two aspects. The first aspect is pro-inflammatory and pro-fibrotic effects (mainly involving MYC, LDLR, and KLF4). MYC is the most typical. It is significantly positively correlated with dendritic cells activated, monocytes, and MCs activated, and significantly negatively linked to T cells gamma delta and MCs resting. In NAFLD, the infiltration of hepatic monocytes and MCs activated are critical for triggering steatohepatitis and liver fibrosis [79]. MYC may disrupt liver immune tolerance, recruit pro-inflammatory cells, and promote disease progression [80]. LDLR and KLF4 are positively correlated with MCs activated and neutrophils, and KLF4 is negatively related to macrophages M2 (anti-inflammatory). This suggests that they may help maintain a chronic inflammatory microenvironment in the liver. The second aspect is immune homeostasis and anti-inflammatory repair (mainly involving CEBPA and TYMS). CEBPA is significantly positively correlated with T cells gamma delta and significantly negatively linked to monocytes. TYMS shows a highly significant negative correlation with MCs activated. Thus, these two genes may play protective roles in NAFLD and help maintain hepatic immune homeostasis. Furthermore, because the distribution of MCs differs significantly between the two clusters and are correlated with all eight NAFLD-LM HGs, special attention should be paid to the role of MCs in the onset of NAFLD. MCs stem from hematopoietic stem cells and mediate inflammation, and they are among the most extensively studied cells in the innate immune system. They contribute to the onset and progression of NAFLD through multiple mechanisms, including promoting microvesicular steatosis in hepatocytes, modulating inflammatory responses and angiogenesis, interacting with the gut microbiota, and cooperating with other immune cells (including T cells and macrophages) to exert immunomodulatory roles [81–85].

The results of scRNA-seq revealed that NR4A2, MYC, LDLR, and KLF4 were predominantly distributed within the following cell clusters, namely central memory T cells, Th17 cells, and KCs. Central memory T cells infiltrate the liver fibrosis region and are accompanied by the release of pro-inflammatory cytokines (e.g., interleukin-17 A (IL-17 A)) [86]. Th17 cells are the largest cell cluster annotated in this study and are a key pro-inflammatory subset of CD4^+^ T cells. Th17 cells secrete the signature cytokine IL-17 A. IL-17 A induces systemic adipose tissue inflammation, drives hepatic triglyceride accumulation and inflammatory infiltration, and promotes liver fibrosis through multiple pathways, including direct activation of HSCs and increasing the expression of transforming growth factor-beta (TGF-β, a pro-fibrotic cytokine) [87, 88]. Another study has shown that the fatty liver microenvironment is conducive to the formation of the CXCR3^+^ Th17 cell population that is sensitive to metabolic stimulation. These cells are more prone to release higher levels of pro-inflammatory factors while promoting the recruitment and activation of macrophages, further exacerbating pathological damage of the liver [89]. KCs, as liver-resident macrophages, are the first responders to initial lipotoxicity and oxidative stress, promoting liver inflammation and fibrosis through multiple pathways, including secretion of pro-inflammatory cytokines and TGF-β [90]. The regulatory network composed of central memory T cells, Th17 cells, and KCs jointly drives the progression from NAFL to fibrosis via inflammation. The enriched expression of NR4A2, MYC, LDLR, and KLF4 in these three cell clusters suggests that they may exert key roles in the regulation of immune cell function in NAFLD.

The model for the eight NAFLD-LM HGs constructed in this study not only has high diagnostic efficacy for NAFLD but also provides a good clinical net benefit for patients. In comparison with other well-studied biomarkers, like Cytokeratin‑18 (a specific marker of hepatocyte apoptosis that helps distinguish NAFL from NASH, AUC = 0.750 or 0.82) and patatin-like phospholipase domain-containing protein 3 (strongly associated with the genetic variation risk for NAFLD, AUC = 0.5537 or 0.596) [91–94], our results still show good predictive performance (AUC = 0.948). Although these biomarkers have potential in NAFLD prediction, their widespread clinical application is limited by factors such as high detection costs and the need for further validation in large, diverse populations [93]. Apart from liver biopsy, the commonly used non‑invasive diagnostic methods for NAFLD are imaging examinations, serum biomarkers and their associated scores. Elevated serum ALT/AST, total bilirubin, or lipid levels usually indicate liver injury or lipid metabolic disorders. However, approximately 80% of individuals with hepatic steatosis do not show abnormal biochemical parameters [93]. The accuracy of imaging examinations and serum biomarker scores varies among different disease types [93, 95]. These non‑invasive diagnostic methods all have limitations. Therefore, it is currently recommended to combine multiple methods, like imaging examinations, serum biomarkers and their scores, and other biomarkers, to improve the diagnostic efficacy for NAFLD [93].

This study has three limitations. First, methodological imperfections are as follows: (1) After the integration of multiple GEO datasets, residual sample heterogeneity bias may still exist. (2) The selection of thresholds for screening LMRGs lacks clear justifications. (3) The machine learning strategy carries a potential risk of overfitting. These issues may affect the reliability of the results. Second, the single‑cell analysis only included three NAFLD patient samples and relied on a single algorithm. This approach may be insufficient to fully elucidate the specific mechanisms through which the hub genes influence NAFLD via immune regulation. Third, the expression trends of some genes were inconsistent between NAFLD patients and mouse. This may be related to model differences, sample sizes, and detection methods, thereby limiting a comprehensive understanding of the regulatory roles of these genes. Although this study confirms the potential of the NAFLD‑LM HGs in the diagnosis and risk stratification of NAFLD, achieving the accuracy, generalizability, and feasibility of their clinical application remains challenging due to detection costs. Future researchers should refine the methodology, cross‑validate results using multiple algorithms, and conduct large‑scale, multi‑center validation in heterogeneous populations. These are critical steps to translate these hub genes from theoretical exploration to clinical application. During validation, combining imaging examinations, serum biomarker scores, and other methods can improve individualized diagnostic performance. In addition, the following experimental schemes may be adopted to further verify the regulatory roles of hub genes. First, adeno-associated virus 8 - thyroxin binding globulin vectors can be used to achieve hepatocyte-specific overexpression or knockdown of the hub gene, thereby validating its in vivo regulatory role in the pathological process of NAFLD. Second, the Seahorse XF Analyzer can be employed to dynamically measure lactate metabolic flux in hepatocytes following perturbation of the target gene, thus defining the metabolic phenotype regulated by the hub gene. Third, chromatin immunoprecipitation sequencing can be performed to verify the binding of the hub gene to its downstream target gene loci and to elucidate the underlying transcriptional regulatory mechanisms.

## Conclusion

NAFLD is a complex disease mediated by metabolic disorders and immune dysfunction. Early identification and treatment of NASH and fibrosis are crucial for improving clinical outcomes in NAFLD patients. This study focused on lactate metabolism and identified eight NAFLD‑LM HGs (CEBPA, MYC, IGFBP1, NR4A2, LDLR, TYMS, FMO1, and KLF4) through bioinformatics analysis. These genes exhibit good diagnostic efficacy for NAFLD and enable risk stratification between early NAFLD and active NASH by revealing differences in the “metabolic‑immune” phenotypes among NAFLD patients. Through subsequent animal experiments, we ultimately identified five genes (LDLR, MYC, IGFBP1, NR4A2, and KLF4) that may exert potential protective effects against NAFLD. Notably, for the functional effects of these genes, complete consensus has not yet been reached across previous studies. Thus, their patterns of effects in NAFLD may be influenced by various factors like disease stage and metabolic microenvironment. Currently, there is no direct evidence to explain how these hub genes are involved in the pathological process of NAFLD through regulating lactate metabolism. This remains one of the unresolved key issues in the present study. Future research should focus on two major directions, namely mechanism elucidation and stability validation, with particular emphasis on the targeted intervention potential of the five protective genes.

## Supplementary Information

Below is the link to the electronic supplementary material.

Supplementary Material 1

Supplementary Material 2

## Data Availability

All datasets utilized in the present research are accessible from the respective online databases. Additional detailed information, including gene names obtained by WGCNA analysis and the four machine learning methods, DEGs identified by consensus clustering, and complete GO terms and KEGG terms, is available in the Supplementary Material.
